# Genomic Profiling of a Human Leukemic Monocytic Cell-Line (THP-1) Exposed to Alpha Particle Radiation

**DOI:** 10.1100/2012/205038

**Published:** 2012-10-11

**Authors:** Vinita Chauhan, Matthew Howland

**Affiliations:** Consumer and Clinical Radiation Protection Bureau, Health Canada, Healthy Environment and Consumer Safety Branch, Ottawa, ON, Canada K1A 0K9

## Abstract

This study examined alpha (**α**-) particle radiation effects on global changes in gene expression in human leukemic monocytic cells (THP-1) for the purposes of mining for candidate biomarkers that could be used for the development of a biological assessment tool. THP-1 cells were exposed to **α**-particle radiation at a dose range of 0 to 1.5 Gy. Twenty-four hours and three days after exposure gene expression was monitored using microarray technology. A total of 16 genes were dose responsive and classified as early onset due to their expression 24 h after exposure. Forty-eight transcripts were dose responsive and classified as late-onset as they were expressed 72 h after exposure. Among these genes, 6 genes were time and dose responsive and validated further using alternate technology. These transcripts were upregulated and associated with biological processes related to immune function, organelle stability and cell signalling/communication. This panel of genes merits further validation to determine if they are strong candidate biomarkers indicative of **α**-particle exposure.

## 1. Introduction

Counterterrorism and national security have become high priority issues worldwide. In spite of the magnitude of threats posed by radiological dispersal devices (RDDs), our capabilities for effectively responding to such a threat remain limited [1]. The majority of the isotopes suspected for use in RDDs are those emitting *α*-particle radiation including Radium-226, Polonium-210, Americium-241, and Plutonium-238 [2]. These isotopes have a very long half-life, emitting their radiation slowly which results in very low continuous dose [2]. All are present in commercial products, in nuclear reactors, and radium-226 occurring in nature. It has been estimated in a simulated RDD scenario using Amercium-241, that of the exposed population, 35% will receive a dose of between 0.05 and 5 Sv of radiation [3, 4]. In a real radiological/nuclear event, the ability to accurately and rapidly estimate the level of exposure to the people at risk is paramount to successful medical intervention. As such, high-throughput methods of radiation detection are desirable. To date, the majority of the radiation detection capabilities have relied on cytogenetic analysis of blood from the exposed individual. However these techniques are time consuming and limited by low throughput. With the widespread use of genomics and proteomics technology in biomarker discovery, the development of a biological assessment tool based on gene markers may prove to be more viable, especially with *α*-particle radiation due to its high damaging capacity.

It has been estimated that the damage received from one alpha particle is ~20 times more damaging than X-rays. Alpha particles have a high linear energy transfer (LET) which are typically 160 keV·*μ*m^−1^ for 2.5 MeV *α*-particles in comparison to 2.0 keV·*μ*m^−1^ for X-rays [5]. High LET *α*-particles create very dense ionizing tracks as they traverse a medium. Therefore, they produce more significant biological effects when compared to equal absorbed doses from low LET radiation, which are more sparsely ionizing [5, 6]. If ingested or inhaled, radionuclides which emit *α*-particles can cause significant damage to sensitive cells and internal human tissue [7]. Alpha particle radiation results in adverse biological effects that can lead to systemic effects and the expression of a wide spectrum of biomolecules (genes, proteins, lipids, and peptides) that may be differentially modulated and therefore can serve as potential biomarkers. Indeed, the naturally occurring *α*-particle emitter radon gas (^222^Rn) has been shown to cause effects and perturbations of the normal state as exemplified by the induction of lung cancer in uranium miners, and further studies with animals have also shown a strong correlation between the exposure of ^222^Rn and the incidence of lung cancer [8, 9]. When inhaled, ^222^Rn gas penetrates into the alveoli, where gas exchange takes place between the lungs and the blood. In general, this can lead to cascade of events that can cause damage to the sensitive cells lining the lungs and to the surrounding nucleated blood cells [10]. This may result in severe cytogenetic damage as a result of multiple ionizations within the DNA structure [11]. A number of *in vitro* and *in vivo* studies have shown that *α*-particle exposure can lead to mutagenic changes including large deletions, frameshifts, and base-change mutations (reviewed 12).

Relatively few studies have examined global gene expression changes following exposure to *α*-particle radiation [12–14]. This study design was chosen with the overarching goal of identifying gene markers of *α*-particle radiation exposure at dose levels that may have relevance to moderate dose whole body exposures. Thus, we sought to explore the merit of genomic profiling strategies in biomarker discovery following *in vitro* exposure of human monocytic cells to *α*-particle radiation. The screening of 40,000 genes in this blood monocytic cell line will allow for the identification of a panel of markers that can be used further for screening *in vivo* and in occupational cohorts. 

## 2. Materials and Methods

### 2.1. Cell Culture

A human monocytic cell line (THP-1) cultured from the blood of a male with acute monocytic leukemia was obtained from American Type Culture Collection (ATCC, Manassas, VA, USA). THP-1 cells were maintained in a humidified incubator (37°C, 5% CO_2_/95% air) in 75 cm^2^ tissue culture flasks (Costar, Cambridge, MA, USA). The cells were grown to confluence for 2-3 days in Royal Park Medical Institute-1640 (RPMI-1640) (Invitrogen Canada, Burlington, ON, Canada) medium, containing 10% fetal bovine serum (FBS) (Sigma-Aldrich Canada, Oakville, ON, Canada). A total of 1.0 × 10^6^ cells were seeded into 5 mL of culture media containing 100 units/mL of penicillin and 100 *μ*g/mL of streptomycin (Invitrogen Canada Inc.). The cells were exposed to *α*-particle radiation at doses ranging from 0.0 (control) to 1.5 Gy, using ^241^Americium (^241^Am) electroplated discs (Eckert and Ziegler Isotope Products Ltd., Valencia, CA, USA) having an activity level of 66.0 kBq ±3% (dose rate of 0.98 ± 0.01 Gy/h, LET of 127.4 ± 0.4 keV/*μ*m). The absorbed dose of *α*-radiation to which cells were exposed was calculated using the GEANT4 v.9.1 Monte Carlo toolkit [15]. For the *α*-particle exposures, cells were cultured in thin Mylar-based plastic dishes (MDs) (Chemplex Industries, Palm City, FL, USA), which allowed penetration of the *α*-particles. Cell viability and apoptotic data are presented in a previous manuscript [16].

### 2.2. RNA Extraction

Twenty-four hours and three days following exposure to *α*-particle radiation or negative control conditions, 5 mL of cell culture were transferred to 15 mL Falcon centrifuge tubes (Invitrogen, Canada) and centrifuged at 200× g for 5 min to pellet the cells. The supernatant was decanted, and the pelleted cells were resuspended in 350 *μ*L of Buffer RLT containing 1%  *β*-Mercaptoethanol (Qiagen's RNeasy Mini kit; Qiagen Inc., Mississauga, ON) and then frozen at −80°C until processed. Frozen THP-1 cells were thawed on ice and mixed well by pipetting. The lysate was transferred directly onto a QIAshredder spin column (Qiagen Inc.), placed in a 2 mL collection tube and centrifuged for 2 min at ~12,000 g. A volume of 350 *μ*L of 70% ethanol was added. Total RNA was then extracted using the RNeasy Mini kit according to the manufacturer's instructions (Qiagen Inc.), with the addition of Qiagen's On-Column RNase-free DNase (Qiagen Inc.) to eliminate any remaining DNA contamination. All total RNA sample concentrations and RNA quality were determined using both an Agilent 2100 Bioanalyzer and RNA Nanochips (Agilent Technologies Canada Inc., Mississauga, ON) and spectrophotometrically using a Nanodrop (Fisher Scientific) (OD ratio of A260 : A280). All extracted RNA samples were determined to be of good quality (RNA Integrity Number = 10) with minimal degradation and stored at −80°C until further analysis. Samples with an RIN value of greater than or equal to 8.0 were deemed to be acceptable for analysis. An input of 200 ng of total RNA was used for whole genome analysis following the Illumina(r) Whole Genome Expression Profiling Assay Guide (11317302 Rev. A). Samples were hybridized on Illumina human-12 v2 RNA BeadChips. BeadChips were imaged and quantified with the Illumina iScan scanner, and data was processed with Illumina GenomeStudio v2010.2.

### 2.3. Statistical Analysis

Data preprocessing was done within GenomeStudio, where the intensities were averaged per probe/gene. Normalization of dataset was conducted in GeneSpring (version GX 11.5). Intensities were normalized to the 25th percentile. Intensities were log2 transformed, and a two-tailed *t*-test was performed. The variance was not assumed to be the same between the groups. Multiple testing using Benjamini and Hochberg false discovery correction was applied to the *P* values in order to obtain robust responding gene targets. 

### 2.4. Quantitative Real Time-Polymerase Chain Reaction (qRT-PCR)

Selected genes identified by microarray analysis displaying statistical significance, with fold changes of 2 or higher and for which primers were validated, were further assessed by qRT-PCR. Total RNA (100 ng) isolated from cells was reverse transcribed into complementary DNA using the RT2 First Strand Kit (SABiosciences Corp., Frederick, Maryland, USA). Gene profiling was performed according to the manufacturer instructions using custom RT2-profiler PCR arrays (SABiosciences Corp.). Reactions were prepared in 96-well plates and performed in duplicate in a spectrofluorometric thermal cycler (Biorad iCycler; Hercules, CA). The relative expression of each gene was determined by using the comparative threshold (Ct) method [13]. Analysis of qRT-PCR expression profiles and statistical analysis of data were performed using the superarray biosciences web portal for data analysis of their products. (SABiosciences http://www.sabiosciences.com/pcr/arrayanalysis.php/).

### 2.5. Pathway Analysis

Significantly expressed genes obtained from the exposure of human monocytic cells to *α*-particle radiation were used for pathway analysis. Gene lists for the dose-dependent genes for both time points were uploaded into data analysis software (Ingenuity Pathway Analysis (IPA), version 7.5; Ingenuity Systems, CA) (IPA, 2005) and used for core analysis with the following settings: Reference Set = Ingenuity's Knowledge Base (genes only) [17, 18]. The same lists were also uploaded as filtered datasets for use in overlaying expression values with the following additional settings: fold-change cutoff = 1.0, *P* value cutoff = 0.05, focus on up- and downregulated identifiers, resolve duplicates = maximum fold change, color nodes by fold change. Core comparison analysis was also run to show the differences in top functions and canonical pathways among the different lists. Functional analysis results were obtained after the analysis was complete. The top high-level and corresponding low-level functions were studied to determine the involved genes and whether those genes increase or decrease the specific function, to make conclusions about the mechanisms in flux after exposure to *α*-particle radiation. Canonical pathway results were obtained after the analysis was complete. Canonical pathway results were customized to display the Benjamini-Hochberg multiple testing correction *P* value to assist in omitting false-positive results from the analysis. The threshold used was *P* value = 0.05 (5% false positive rate). Canonical pathways that had a *P* value of 0.05 or less were further studied to determine the genes that were regulated from these datasets, and how these genes specifically affect the canonical pathway. Networks were used to further corroborate the functional analysis and canonical pathway results and to provide insight into any regulatory mechanisms. Networks were also used to view the molecular connections between the genes of interest to determine if they collectively share common biological functions when working together.

## 3. Results

### 3.1. Gene Profiling Twenty-Four Hours after Exposure

To mine for reliable genes, all differentially expressed genes were filtered on flagged spots that were of poor quality, a 1.5-fold change cut-off and a *P* value <0.05. A total of 41 genes were shown to be expressed solely at the low dose of radiation (0.5 Gy, *P* < 0.05, |FC| > 1.5) (data not shown), and all of these transcripts were upregulated. A total of 21 genes (*P* < 0.05, |FC| > 1.5) were exclusively expressed at the medium (1.0 Gy) and high (1.5 Gy) dose, and all of these genes were upregulated (data not shown). Only 16 genes were shown to be dose-responsive (Table 1), and expression of these genes was observed to be higher relative to unexposed cells. Strong expressors with high fold changes were observed for transcripts *TRIB3*, *SPP1*, and *CD14 *(>3 fold at the highest dose).

### 3.2. Gene Profiling Three Days Following Exposure

As previous work from our laboratory [16] has shown an apoptotic response to occur 4 days subsequent to *α*-particle exposure, in this study gene expression changes were monitored at three days after exposure, before cells proceeded to undergo apoptosis. Significantly more genes were differentially expressed at this time point in comparison to the 24 h time point. Similar to the 24 h time point all genes listed in the Tables are statistically significant and with fold changes >1.5. At the low dose of radiation (0.5 Gy) a total of 62 genes were differentially expressed (*P* < 0.05, |FC| > 1.5), 55% of these genes were upregulated, and 45% were downregulated (data not shown). At the low and medium dose of radiation, a total of 50 genes were differentially expressed and 33 of these were upregulated and 17 were downregulated (data not shown). A total of 48 genes were expressed at all three doses (Table 2). Fold changes for these genes at the highest dose of radiation tested ranged from 5-fold to −2.5-fold. High expressors included *CD14*, *SPP1*, *TRIB3*; these genes were also shown to be expressed at 24 h after *α*-particle exposure. Genes which were highly downregulated included *INSIG1*, *KIT*, *SC4MOL,* and *SCD*.

### 3.3. Dose- and Time-Responsive Genes

**Figure 1:**
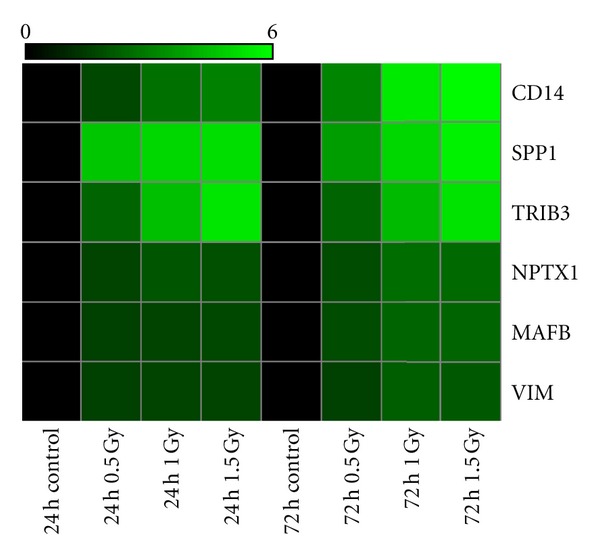
Heat map of dose- and time- responsive genes and corresponding fold changes from the microarray data. These genes were statistically significant (*P* < 0.05) at all doses and time points tested on an *n* = 5 biological replicates.

A total of 6 genes that were observed to be expressed at the 24 h time point were also shown to respond 3 days following *α*-particle exposure (Table 3). These 6 genes may be highly radiation-specific and can be classified as dose- and time-responsive genes. All six of these genes were significantly upregulated. Three of these transcripts had expression levels of 5 fold or higher 3 days after exposure the genes include *TRIB3*, *CD14,* and *SPP1*. The 6 genes that exhibited time- and dose-dependant trends were further used to create a heat map analysis of all treatment groups (Figure 1). The figure further highlights the linear dose and time-response trends exhibited by the majority of these genes. 

### 3.4. qRT-PCR Gene Validation

For a selected few of the dose-time responsive genes and transcripts that were expressed at all doses at the 3 day time-point, qRT-PCR validation was performed. As shown in Table 4, all genes that exhibited a significant dose- and time-response were also observed to exhibit a similar trend using qRT-PCR. A comparable pattern of expression was also observed for the genes (*INSIG1*, *KIT*, *S100P*, *ORM1*) that were dose-responsive but not time-responsive using the two methodologies. Overall, these results show analogous responses between the two technologies.

### 3.5. Pathway Analysis

The dose-responsive genes expressed at 24 h and 72 h were used to conduct pathway analysis of the gene sets. Sixteen dose-responsive genes that were differentially expressed at the 24 h time point were inputted into the IPA analysis tool; the results are summarized in Table 5. The top canonical pathways for these gene sets included toll-like receptor signaling and MIF regulation of innate immunity. Only one network obtained a high score and it was associated with carbohydrate/lipid metabolism and small molecule biochemistry. The biofunctions of these genes in relation to disease and disorders were respiratory disease, genetic disorder, and inflammatory disease. Molecular and cellular functions included cell-cell signaling and interaction, cellular development, and cellular growth and proliferation. The physiological functions were in relation to tissue development and immune cell trafficking and nervous system development and function. The top genes associated with these pathways were *SPP1*, *TRIB3*, *VCAN*, *CD14*, *ATF5,* and *KCNG1*; all were upregulated in expression following exposure to *α*-particles.

Forty-eight genes that were expressed at the 72 h time point were inputted into the IPA analysis tool. The data showed similar responding pattern of effects to the genes obtained 24 h after *α*-particle exposure (Table 6). The top networks associated with this gene set included lipid metabolism, cell-to-cell signaling interaction, cell death, and developmental disorder. The top biofunctions in relation to diseases and disorders were cancer, respiratory disease, and inflammatory response. Molecular and cellular functions of these genes were in relation to lipid metabolism, molecular transport, and cellular movement. In terms of physiological system development and function the top bio-functions of the genes were in relation to haematological system development and function, immune cell trafficking, lymphoid tissue structure and development. The top canonical pathways associated with these genes were IL-6 signaling, p53 signaling, and airway pathology in chronic obstructive pulmonary disease. The genes associated with these pathways included *SPP1*, *CD14*, *TRIB3*, *S100P*, *S100A9*, *LYZ*, *IL8*, *PLIN2*, *S100A8,* and *INSIG1*. All were upregulated in expression with the exception of *INSIG1* which was downregulated by ~2-fold following *α*-particle irradiation.

## 4. Discussion

In this study the biological effects of *α*-particle radiation at the genome level were monitored at two time points and three doses with the goal to identify potential gene targets that are modulated with the radiation exposure. These changes were monitored in a human monocytic cell line which may be more relevant to biomarker discovery. This cell line serves as a good model for use in an initial screening to identify potential biomarkers of *α*-particle radiation exposure. However, further studies would be required to confirm the validity of these markers using *in vivo* studies and investigations based on using occupational cohorts. 

The results from this study show that *α*-particles elicit strong responses at the transcript level in THP-1 cells, and these responses vary with time point and dose of radiation. Although gene modulations were obtained independently at each of the doses tested, only those responses that were elicited at all doses may be important candidate biomarkers that would require further validation. Gene expression changes were monitored at 24 h and 72 h following exposure. Of the ~48,000 transcripts that were screened for expression, only 16 genes were differentially expressed relative to untreated samples at the 24 h time point at all three doses tested. Three days following *α*-particle exposure a greater number of genes were expressed, with a total of 48 genes showing dose-responsive trends. Analysis of theses gene sets showed networks related to cell-to-cell signaling and interaction, molecular transport, lipid/carbohydrate, organism injury, and abnormalities. The disease and disorders related to these genes were respiratory disease, cancer, and inflammatory disease. Top toxicological pathways in relation to disease include airway pathology in chronic obstructive pulmonary disease. Among these 48 genes, only 6 were also present 24 h after exposure. It is these six genes that may warrant further studies. Gene ontology analysis of these six genes (*TRIB3*, *CD14*, *VCAN*, *SPP1*, *NPTX1*, *MAFB*, *VIM*) suggests that they are associated with functions related to immune function, organelle stability, and cell signalling/communication and pathways associated with p53 signaling and IL-6 signaling (Table 7). Although not within the scope of this study, further studies are required to determine the specificity of this response to this radiation type in comparison to other radiation types. 

Studies on *α*-particle exposure and gene expression changes are limited; however a few studies have obtained similar findings. A recent study examined gene expression changes in human peripheral blood lymphocytes from two patients exposed ex vivo to 6.78 MeV mean energy alpha particles from extracellular ^211^At source [14]. Two hours after exposure, at doses ranging from 0.05 to 1.6 Gy, eight genes displayed a sustained up- or downregulation (*CD36*, *HSPA2*, *MS4A6A*, *NFIL3*, *IL1F9*, *IRX5*, *RASL11B,* and *SULT1B1*). A comparison of these results with our study shows a very similar grouping of genes, despite the use of different exposure conditions, cells, and time points. Both of the studies have obtained genes associated within the interleukin family of signaling molecules, cell cycle/division processes, proliferation, and differentiation. In line with this, Roudkenar et al. [12] investigated the effect of particles and lithium nuclei generated by boron neutron capture reaction on mouse liver epithelial and Kupffer cells. Of the 6000 genes that were examined, 68 showed differential expressions compared to the nonirradiated controls. Some of the genes that were differentially expressed were involved in cell cycle regulation, intracellular transport, and fatty acid metabolism. In another study Seidl et al. [19] exposed gastric cancer cells to *α*-particle irradiation and monitored global changes in gene expression. The authors identified majority of the significantly upregulated genes to be related to signal transduction processes. Overall results from these studies show that similarities exist between investigations in terms of the functional classification of the differentially expressed transcripts. This is promising as the six time- and dose-responsive genes merit further investigation to assess their potential to be biomarkers.

In summary, this study showed that *α*-particles exert modulations upon the transcriptional activity of human leukemic monocytic cells. These modulations varied with dose and time subsequent to exposure. A subset of genes were identified that were expressed at low-to-moderate doses of radiation in a time- and dose-dependant manner. These genes broadly fall into the family of immune/signaling molecules and could be reliable candidate biomarkers of *α*-particle radiation exposure. Although this study has verified the use of genomic technology for biomarker discovery, further validation of these results required using *in vivo* modeling and *in vitro* monocyte isolation from individuals. Future studies will validate these responses over a wider dose ranges and using alternate radiation types.

## Figures and Tables

**Table:** (a) 24 h

	0.5 Gy	1.0 Gy	1.5 Gy
Number of Transcripts	**38**	**84**	**163**
Common Amongst All Doses (%)	**15** (39)	**15** (18)	**15** (9)
Exclusive (%)	13 (34)	10 (12)	87 (53)
Upregulated (%)	**38 **(100)	**81 **(96)	**147 **(30)
Common Amongst All Doses (%)	15 (39)	15 (18)	15 (51)
Exclusive (%)	13 (34)	10 (5)	74 (18)
Downregulated (%)	**N/A **(0)	**3**(4)	**16 **(10)
Common Amongst All Doses (%)	N/A	3 (100)	3 (19)
Exclusive (%)	N/A	0 (0)	13 (81)

**Table:** (b) 72 h

	0.5 Gy	1.0 Gy	1.5 Gy
Number of Transcripts	**143**	**152**	**190**
Common Amongst All Doses (%)	**47** (33)	**47 **(31)	**47 **(25)
Exclusive (%)	82 (57)	25 (16)	57 (30)
Upregulated (%)	**110 **(77)	**24 **(16)	**50 **(26)
Common Amongst All Doses (%)	15 (14)	15 (63)	15 (8)
Exclusive (%)	82 (75)	2 (8)	23 (12)
Downregulated (%)	**33 **(23)	**128 **(84)	**140 **(74)
Common Amongst All Doses (%)	32 (97)	32 (25)	32 (23)
Exclusive (%)	1 (3)	23 (18)	34 (24)

**Table 2:** Expression profiles of genes that were shown to be statistically significant 72 h after alpha-particle irradiation at all doses. Statistics based on an *n* = 5 biological replicates.

Accession no.	Symbol	FC-D1	Pv	FC-D2	Pv	FC-D3	Pv
NM_001040021.1	CD14	3.15	0.00	5.50	0.00	5.89	0.00
NM_000582.2	SPP1	3.72	0.00	4.98	0.00	5.68	0.04
NM_021158.3	TRIB3	2.43	0.00	4.32	0.00	5.34	0.00
NM_000607.1	ORM1	2.49	0.00	3.62	0.00	3.67	0.00
NM_005980.2	S100P	2.28	0.00	3.25	0.00	3.44	0.00
NM_002965.2	S100A9	2.13	0.00	2.43	0.00	2.48	0.00
NM_002522.2	NPTX1	1.82	0.00	2.57	0.00	2.48	0.00
NM_006216.2	SERPINE2	1.75	0.00	2.51	0.00	2.44	0.00
NM_001122.2	ADFP	1.88	0.02	2.29	0.00	2.43	0.00
NM_005461.3	MAFB	1.86	0.01	2.36	0.00	2.41	0.00
NM_001995.2	ACSL1	1.55	0.00	2.34	0.00	2.34	0.00
NM_001375.2	DNASE2	1.66	0.01	2.05	0.00	2.22	0.00
NM_002964.3	S100A8	1.87	0.00	2.19	0.00	2.21	0.00
NM_000239.1	LYZ	2.08	0.00	2.26	0.00	2.19	0.00
NM_015869.4	PPARG	1.68	0.00	2.13	0.00	2.17	0.00
NM_019058.2	DDIT4	1.57	0.00	2.15	0.00	2.15	0.00
NM_000104.2	CYP1B1	1.82	0.00	2.15	0.00	2.13	0.00
NM_003380.2	VIM	1.56	0.00	2.20	0.00	2.10	0.00
NM_000584.2	IL8	2.01	0.01	2.07	0.00	2.07	0.01
NM_000632.3	ITGAM	1.83	0.00	2.07	0.00	2.06	0.00
NM_006366.2	CAP2	1.51	0.00	1.92	0.00	2.03	0.00
NM_002928.2	RGS16	1.54	0.00	1.66	0.00	1.98	0.00
NM_003376.4	VEGFA	1.54	0.00	2.03	0.00	1.97	0.00
NM_003916.3	AP1S2	1.60	0.00	2.01	0.00	1.97	0.00
NM_004811.1	LPXN	1.50	0.00	2.18	0.00	1.94	0.00
NM_174918.2	C19orf59	1.55	0.00	2.12	0.00	1.92	0.00
NM_013363.2	PCOLCE2	1.68	0.00	1.96	0.00	1.90	0.00
NM_000889.1	ITGB7	1.68	0.00	1.91	0.00	1.89	0.00
NM_001629.2	ALOX5AP	1.57	0.00	1.98	0.00	1.81	0.00
NR_002203.1	FTHL8	1.51	0.03	1.67	0.00	1.67	0.00
NM_018004.1	TMEM45A	1.53	0.01	1.92	0.00	1.60	0.00
NM_000265.4	NCF1	1.53	0.01	1.55	0.01	1.55	0.00
NM_003583.2	DYRK2	0.57	0.02	0.64	0.01	0.63	0.01
NM_152764.1	C16orf73	0.64	0.00	0.65	0.00	0.62	0.00
NM_001821.3	CHML	0.54	0.01	0.61	0.00	0.61	0.00
NM_002130.6	HMGCS1	0.54	0.01	0.56	0.00	0.61	0.00
NM_005640.1	TAF4B	0.56	0.01	0.66	0.01	0.61	0.03
NM_001005291.1	SREBF1	0.61	0.01	0.62	0.00	0.59	0.00
NM_001093772.1	KIT	0.58	0.00	0.60	0.00	0.57	0.00
NM_000527.2	LDLR	0.60	0.03	0.59	0.00	0.56	0.00
NM_000820.1	GAS6	0.62	0.02	0.64	0.00	0.54	0.00
NM_033445.2	HIST3H2A	0.48	0.02	0.58	0.04	0.54	0.02
NR_002771.1	DLEU2L	0.57	0.01	0.60	0.00	0.53	0.00
XR_001514.1	DLEU2	0.64	0.01	0.60	0.00	0.51	0.00
NM_005063.4	SCD	0.56	0.01	0.57	0.00	0.49	0.00
NM_001017369.1	SC4MOL	0.54	0.01	0.53	0.01	0.48	0.00
NM_000222.1	KIT	0.50	0.01	0.53	0.00	0.42	0.00
NM_198336.1	INSIG1	0.54	0.03	0.44	0.00	0.41	0.00

**Table 3:** Expression profile of genes validated using qRT-PCR. Fold changes and associated *P* values are indicated for both time points and all doses.

Accession no.	Symbol	0.5 Gy				1.0 Gy				1.5 Gy			
		FC-T1	Pv	FC-T2	Pv	FC-T1	Pv	FC-T2	Pv	FC-T1	Pv	FC-T2	Pv
NM_000582	SPP1	10.865	1.2*E* − 05	16.3775	0.0164	15.48	4*E* − 04	21.56	0.0456	15.18	3*E* − 06	22.026	0.163
NM_000591	CD14	2.349	0.0011	7.8024	0	4.927	0.001	10.66	8*E* − 06	6.215	2*E* − 04	11.534	0.01
NM_002522	NPTX1	1.485	0.07025	3.1824	4*E* − 05	2.39	6*E* − 04	3.523	2*E* − 05	2.599	8*E* − 05	3.2643	0.008
NM_003380	VIM	1.2647	0.30111	2.3705	0.0161	1.248	0.271	2.965	0.0008	1.622	0.028	3.1475	0.004
NM_005461	MAFB	1.4789	0.15033	4.4038	0.0001	2.139	0.012	5.656	6*E* − 05	2.968	6*E* − 04	4.9452	0.017
NM_021158	TRIB3	2.0386	0.01668	3.2229	0.0002	5.729	3*E* − 04	5.373	0.0003	6.746	4*E* − 04	5.7073	3*E* − 04
NM_005542	INSIG1	−1.58	0.00271	−2.01	0.0228	−1.36	0.013	−2.56	0.001	−1.75	0.002	−2.89	6*E* − 04
NM_000222	KIT	−1.18	0.20533	−2.515	0.0101	−1.09	0.411	−3.22	0.0014	−1.49	0.014	−3.35	0.001
NM_005980	S100P	0.9565	0.66173	3.9682	4*E* − 06	1.371	0.067	4.48	2*E* − 06	1.632	0.002	4.5418	2*E* − 05
NM_000607	ORM1	1.0251	0.83691	4.5517	0.0003	1.406	0.048	6.452	0.0003	1.638	0.02	6.4402	5*E* − 04

**Table 4:** Expression profiles of genes that were observed to be dose- and time-responsive using microarray technology.

Symbol	0.5 Gy				1.0 Gy				1.5 Gy			
	FC-T1	Pv	FC-T2	Pv	FC-T1	Pv	FC-T2	Pv	FC-T1	Pv	FC-T2	Pv
CD14	1.77	0.00	3.15	0.00	2.63	0.00	5.50	0.00	3.08	0.00	5.89	0.00
MAFB	1.59	0.00	1.86	0.01	1.63	0.00	2.36	0.00	1.77	0.00	2.41	0.00
NPTX1	1.62	0.00	1.82	0.00	1.98	0.00	2.57	0.00	1.90	0.00	2.48	0.00
SPP1	4.61	0.00	3.72	0.00	5.05	0.00	4.98	0.00	5.17	0.00	5.68	0.04
TRIB3	2.40	0.00	2.43	0.00	4.46	0.00	4.32	0.00	5.39	0.00	5.34	0.00
VIM	1.56	0.00	1.56	0.00	1.62	0.00	2.20	0.00	1.62	0.00	2.10	0.00

**Table 5:** Canonical functions and networks of dose-responsive genes that were differentially expressed 24 h after irradiation

Category	Score	*P* value
Networks		
Carbohydrate Metabolism, Lipid Metabolism, Small Molecule Biochemistry	21	N/A
Diseases and Disorders		
Genetic Disorder	N/A	4.45*E* − 02
Inflammatory Response	N/A	3.85*E* − 02
Respiratory Disease	N/A	4.40*E* − 02
Molecular and Cellular Functions		
Cell-To-Cell Signalling and Interaction	N/A	4.60*E* − 02
Cellular Development	N/A	4.80*E* − 02
Cellular Growth and Proliferation	N/A	3.66*E* − 02
Physiological System Development and Function		
Tissue Development	N/A	4.25*E* − 02
Haematological System Development and Function	N/A	4.80*E* − 02
Immune Cell Trafficking	N/A	4.60*E* − 02
Top Canonical Pathways		
MIF Regulation of Innate Immunity	N/A	2.05*E* − 02
Toll-like Receptor Signalling	N/A	2.50*E* − 02

**Table 6:** Canonical Functions and networks associated with the dose responsive genes that were differentially expressed 72 h after irradiation.

Category	Score	*P* value
Networks		
Lipid Metabolism, Molecular Transport, Small Molecule Biochemistry	36	N/A
Carbohydrate Metabolism, Cell-To-Cell Signalling and Interaction, Cell Death	14	N/A
Developmental Disorder, Organism Injury and Abnormalities, Infectious Disease	8	N/A
Diseases and Disorders		
Cancer	N/A	3.39*E* − 03
Haematological Disease	N/A	3.39*E* − 03
Genetic Disorder	N/A	3.39*E* − 03
Inflammatory Response	N/A	3.39*E* − 03
Respiratory Disease	N/A	2.38*E* − 03
Molecular and Cellular Functions		
Lipid Metabolism	N/A	3.39*E* − 03
Molecular Transport	N/A	3.39*E* − 03
Small Molecule Biochemistry	N/A	3.39*E* − 03
Cell Death	N/A	3.39*E* − 03
Cellular Movement	N/A	3.39*E* − 03
Physiological System Development and Function		
Haematological System Development and Function	N/A	3.39*E* − 03
Immune Cell Trafficking	N/A	3.28*E* − 03
Lymphoid Tissue Structure and Development	N/A	3.33*E* − 03
Tissue Development	N/A	3.39*E* − 03
Top Canonical Pathways		
IL-6 Signalling	N/A	1.09*E* − 02
p53 Signalling	N/A	1.14*E* − 02
Airway Pathology in Chronic Obstructive Pulmonary Disease	N/A	1.35*E* − 02

**Table 7:** The biological functions associated with the time- and dose-responsive genes as determined using Ingenuity Pathway Analysis software.

Accession number	Gene	Function
NM_001040021.1	CD14	Immune function, cell immunity differentiation
NM_005461.3	MAFB	Transcription factor, hematopoiesis,
NM_002522.2	NPTX1	Neural pathways, immune function
NM_000582.2	SPP1	Immune function, antiapoptosis,
NM_021158.3	TRIB3	NF-kappa B signalling, AKT 1 signalling, apoptosis
NM_003380.2	VIM	Cell resilience to mechanical stress, organelle stability,

## Abbreviations

(^241^Am): Americium
(^222^Rn): Radon
(^226^Ra): Radium
(^210^Po): Polonium
(MD): Mylar Based plastic dishes
(FBS): Fetal bovine serum
(TBS): Triphosphate buffered saline
(TST): TBS serum triton
(PBS): Phosphate buffered saline
(*α*): Alpha
(MD): Mylar dish
(RPMI): Royal park medical institute
(ANOVA): Analysis of variance
(qRT-PCR): Quantitative real-time polymerase chain reaction
(Ct): Compartive threshold
(FC): Fold change
(RDDs): Radiological Dispersal Devices
(LET): Linear Energy Transfer
(IPA): Ingenuity Pathway Analysis.

## References

[1] ICRP (2005). Protecting people against radiation exposure in the event of a radiological attack. *Annals of the ICRP*.

[2] Ferguson C, Potter W (2004). *The Four Faces of Nuclear Terrorism*.

[3] Arutyunian R, Bolshov L, Pavlovskiy O, Khripunov I, Bolshov L, Nikonov D (2007). Radiological terrorism: threat, priorities in prevention, and minimization of consequences. *Social and Psychological Effects of Radiological Terrorism*.

[4] Kelly H http://www.fas.org/ssp/docs/kelly_testimony_030602.pdf.

[5] Johns HE, Cunningham JR (1983). *The Physics of Radiology*.

[6] Cember H (1996). *Introduction to Health Physics*.

[7] Al-Affan IAM, haque AKMM (1989). Local energy deposited for alpha particles emitted from inhaled radon daughters. *Physics in Medicine and Biology*.

[8] Stather JW (2004). Dosimetric and epidemiological approaches to assessing radon doses—can the differences be reconciled?. *Radiation Protection Dosimetry*.

[9] Al-Zoughool M, Krewski D (2009). Health effects of radon: a review of the literature. *International Journal of Radiation Biology*.

[10] National Research Council (1999). *Health Effects of Exposure to Radon (BEIR VI)*.

[11] Goodhead DT (1994). Initial events in the cellular effects of ionizing radiations: clustered damage in DNA. *International Journal of Radiation Biology*.

[12] Roudkenar MH, Li L, Baba T (2008). Gene expression profiles in mouse liver cells after exposure to different types of radiation. *Journal of Radiation Research*.

[13] Azzam EI, De Toledo SM, Little JB (2003). Expression of CONNEXIN43 is highly sensitive to Ionizing radiation and other environmental stresses. *Cancer Research*.

[14] Turtoi A, Brown I, Schläger M, Schneeweiss FHA (2010). Gene expression profile of human lymphocytes exposed to 211at *α* particles. *Radiation Research*.

[15] Beaton LA, Burn TA, Stocki TJ, Chauhan V, Wilkins RC (2011). Development and characterization of an in vitro alpha radiation exposure system. *Physics in Medicine and Biology*.

[16] Chauhan V, Howland M, Chen J, Kutzner B, Wilkins RC (2011). Differential effects of alpha-particle radiation and x-irradiation on genes associated with apoptosis. *Radiology Research and Practice*.

[17] http://www.ingenuity.com/.

[18] http://www.ingenuity.com/.

[19] Seidl C, Port M, Apostolidis C (2010). Differential gene expression triggered by highly cytotoxic *α*-emitter-immunoconjugates in gastric cancer cells. *Investigational New Drugs*.

